# Novasomal Gel for Enhanced Dermal Delivery and Antibacterial Efficacy of Cinnamic Acid

**DOI:** 10.3390/molecules31132277

**Published:** 2026-06-29

**Authors:** Rana Alanazi, Shahad Althumali, Abeer Albalawi, Ghala Alqubaydhi, Mona Qushawy, Ayman Salama, Mona F. Arafa, Helal F. Hetta, Yasmin N. Ramadan, Yasmin Mortagi, Ghareb M. Soliman

**Affiliations:** 1 PharmD Program, Faculty of Pharmacy, University of Tabuk, Tabuk 71491, Saudi Arabia; 2 Department of Pharmaceutics, Faculty of Pharmacy, University of Tabuk, Tabuk 71491, Saudi Arabia; mqushawy@ut.edu.sa (M.Q.); agrawan@ut.edu.sa (A.S.); marafa@ut.edu.sa (M.F.A.); 3 Division of Microbiology, Immunology and Biotechnology, Department of Natural Products and Alternative Medicine, Faculty of Pharmacy, University of Tabuk, Tabuk 71491, Saudi Arabia; hhussen@ut.edu.sa; 4 Department of Microbiology and Immunology, Faculty of Pharmacy, Assiut University, Assiut 71515, Egypt; yasmine_mohamed@pharm.aun.edu.eg; 5 Department of Pharmaceutics, Faculty of Pharmacy, Sinai University, Alarish North Sinai 45511, Egypt; yasmin.mohamed@su.edu.eg

**Keywords:** cinnamic acid, novasomes, bacterial skin infection, skin permeability, antibacterial efficacy

## Abstract

While bacterial skin infections are highly prevalent worldwide, their eradication with conventional topical medications remains highly challenging. Cinnamic acid (CA) is a naturally occurring molecule with interesting antibacterial properties, but its efficacy is hindered by poor aqueous solubility and skin permeability. To overcome these challenges, CA was encapsulated within novasomes, which are multilamellar vesicles composed of fatty acids, cholesterol, and nonionic surfactants. The novasomes were optimized using a 2^3^ factorial design and the optimized formulation was incorporated in a carbopol gel base and evaluated for spreadability, rheological properties, drug release, ex vivo skin permeation and deposition, and antibacterial efficacy. The optimized novasomes featured desirable properties, including high drug entrapment (94.75 ± 0.05%), nanometric particle size (123.80 ± 1.44 nm), and negative zeta potential (−36.63 ± 0.61 mV). CA novasomal gel exhibited shear-thinning behavior, coupled with thixotropic properties. It also achieved approximately 1.7-fold higher flux through rat skin compared with the free CA gel. Moreover, the novasomes showed a two-fold reduction in the minimum inhibitory concentration of the drug against *E. coli* compared with the drug suspension. These findings support the potential of CA novasomal gel to enhance its antibacterial activity and skin permeability, making it a promising approach for topical delivery of this naturally occurring compound.

## 1. Introduction

Skin and soft tissue infections (SSTIs) are among the most common infectious diseases worldwide, making them a significant global health concern [1,2]. They include a broad spectrum of bacterial infections involving the skin, subcutaneous tissue, fascia, and muscles. These infections range from mild superficial infections to severe invasive diseases. The most common SSTIs include impetigo, which is a superficial contagious infection characterized by intra-epidermal vesicles and golden-yellow crusts; folliculitis, furuncles, and carbuncles, which originate from infected hair follicles; cutaneous abscesses, which are shown as localized collections of pus; erysipelas, an acute infection of the upper dermis with well-demarcated erythematous lesions; and cellulitis, which is a diffuse infection of the deep dermis and subcutaneous tissues characterized by redness, warmth, swelling, and pain [3].

Recent epidemiological studies have indicated that the incidence rate of SSTIs was approximately 77.5 cases per 1000 person-years, with higher rates recorded for elderly and comorbid patients [2]. The total hospital admissions due to these infections have increased by 29% during the period between 2000 and 2004 [4]. During the period between 1998 and 2004, *S. aureus* was found to be the primary causative organism of SSTIs in North America [5]. The treatment of these infections usually relies on antimicrobial agents, including topical and oral drugs for mild cases, while intravenous and surgical intervention are used for more severe cases [6]. However, bacterial resistance to antibiotics limits the efficacy of these treatment modalities. Poor drug penetration into deeper skin layers, in addition to bacterial persistence within follicular and dermal locations, are other factors that may result in the poor efficacy of antimicrobial agents [7,8]. These challenges result in a growing demand for advanced drug delivery systems to improve the efficacy of antimicrobial therapy and increase their delivery to deeper skin layers [8].

Cinnamic acid (CA) is a naturally occurring molecule in many plants, especially the bark of cinnamon trees (from the *Cinnamomum* genus) and other parts of plants like balsam, resins, and fruits. It contributes to the smell and taste of cinnamon, giving it a distinctive sweet and spicy smell [9]. CA has a wide range of pharmacological effects, including antidiabetic, neuroprotective, antifungal, anti-inflammatory, antioxidant, and anticancer effects [9]. Additionally, it has notable antimicrobial activity, particularly against pathogenic bacteria. Recent studies have clarified its mechanisms of action, illustrating that it could be a good natural antibacterial agent. These mechanisms include disruption of the bacterial cell membranes, inhibition of ATPase activity, and interference with biofilm formation and quorum sensing. These mechanisms impair bacterial structure and function, which support its potential use as an antimicrobial agent [10,11].

CA topical delivery faces several challenges, which limit achieving its full clinical potential. For instance, poor aqueous solubility reduces its penetration through the skin following topical application, reducing its ability to reach and eradicate bacterial infections in deep skin layers. Additionally, poor stability after exposure to UV light leads to loss of activity, potentially forming harmful degradation products [12,13,14,15,16].

Drug delivery to deep layers of the skin where bacterial infections reside is a prerequisite for effective topical use of antibacterial agents [17]. However, this is confronted by the stratum corneum, which forms the main barrier to drug penetration through the skin [18]. Several approaches and drug delivery strategies have been explored to overcome this limitation. These approaches included the use of physical and chemical permeation enhancers, drug loading within innovative drug delivery systems such as metallic, lipidic, and polymeric nanoparticles, and others [19].

Among the explored innovative drug carriers, lipid-based vesicular systems have shown great promise to effectively deliver the loaded drugs to deep tissue layers and overcome the skin barrier properties [20]. The conventional vesicular systems, such as liposomes, suffer from poor penetration through the skin, leading to drug confinement within the superficial skin layers, thus limiting drug efficacy [21]. To mitigate this limitation, the literature shows several innovative drug delivery systems that are under active investigation, such as transferosomes, transethosomes, binary ethosomes, and novasomes [22,23,24]. These carriers usually have improved membrane fluidity compared to the conventional liposomes due to the inclusion of edge activators such as surfactants, leading to improved drug permeation through the skin.

Novasomes are multilamellar vesicles with multiple bilayers and large internal aqueous cores. Unlike liposomes, which are mainly composed of phospholipids, novasomes are composed of fatty acids, cholesterol, and nonionic surfactants (e.g., sorbitan esters). The inclusion of surfactants makes the novasomes highly flexible, with improved physical and chemical stability. They are therefore considered an enhanced form of liposomal or niosomal structures [25,26,27]. As such, novasomes have demonstrated a promising potential for the encapsulation and delivery of a wide range of drugs and vaccines. Their unique structure allows for improved drug loading, controlled release profiles, and enhanced permeation across biological barriers, making them attractive carriers for pharmaceutical applications [28,29,30,31]. Despite this interesting potential of novasomes, the literature shows that their use to overcome CA limitations and improve its antibacterial efficacy remains largely underexplored.

This study aimed to develop and characterize CA-loaded novasomes to enhance its skin permeability, control its release, and improve its antibacterial efficacy. A 2^3^ factorial design was utilized to optimize the formulations. The optimal formulation was incorporated into a carbopol gel and subjected to detailed evaluation using several techniques. The ex vivo skin permeability was assessed using rat abdominal skin membrane. The potential of this preparation to enhance the drug in vitro antibacterial efficacy was also evaluated.

## 2. Results and Discussion

A 2^3^ factorial design examines three independent factors each at two levels, resulting in eight experimental runs (Table 1). It evaluates the main effects and interactions efficiently, making it ideal for optimizing pharmaceutical formulations and processes. It is advantageous in reducing costs while providing valuable insights during pharmaceutical formulation development. Additionally, the results are analyzed using ANOVA or regression analysis for statistical significance [32]. The CA-loaded novasomes were prepared by the ethanol injection method and evaluated for percentage drug entrapment (EE%), particle size (PS), polydispersity index (PDI), and zeta potential (ZP), with the results being presented in Table 1.

Oleic acid is commonly used in novasomes as the free fatty acid component due to its known ability to modify their structure, membrane flexibility, and permeability. Additionally, it was also shown to act as a penetration enhancer, enabling the loaded drug to reach deep skin layers [33,34]. Span 60 (SP60) and Tween 80 (TW80) were chosen as the nonionic surfactants of the vesicles due to their different hydrophilic lipophilic balances (HLB) values of 4.7 and 15, respectively. HLB was previously shown to influence the properties of novasomes such as EE%, PS, ZP, drug release profiles, and in vivo performance [33,35].

### 2.1. Influence of Independent Variables on EE% (Y1)

**Figure 1:**
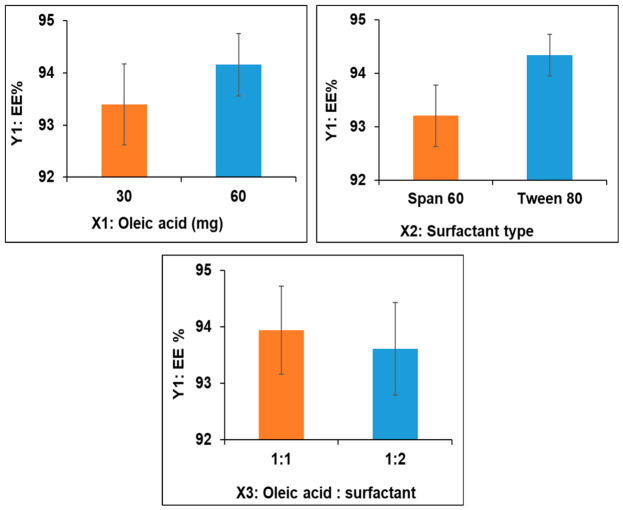
EE% of the novasomes as a function of the tested independent variables.

EE% refers to the drug concentration successfully loaded into a drug carrier relative to the total drug concentration initially used during preparation. High EE% has a significant influence on the safety and efficacy of vesicular drug carriers as it maximizes the drug/excipients ratio, limiting the unwanted excipient effects [36]. The EE% of CA in the prepared novasomes ranged from 92.59 ± 0.25% to 94.75 ± 0.05% and was found to increase with the increase in oleic acid amount (Table 1, Figure 1). Previous studies showed that moderate concentrations of oleic acid increased EE% by increasing the flexibility of the vesicular membrane, which improved drug encapsulation. This was attributed to the unsaturated structure of oleic acid, which disrupted the tight packing of lipid bilayers, allowing more drug molecules to be encapsulated [37].

As for the effect of surfactant type on EE%, TW80 resulted in a slightly higher EE% compared with SP60. These findings were unexpected and were in contrast with previous studies showing the opposite for lipophilic drugs such as CA [33,38]. SP60 is a lipophilic nonionic surfactant with a low HLB value (4.7), and characterized by a long saturated alkyl chain and a high phase transition temperature (~53 °C) [39]. This leads to an increase in the rigidity of the vesicle bilayer, and, consequently, higher encapsulation of lipophilic drugs. For instance, SP60 niosomes had higher ondansetron EE% compared to those of SP80, TW80, and TW20 due to the lower HLB value of SP60 and saturated alkyl chain, which favor the entrapment of lipophilic drugs [38,40]. The reason for the slightly higher EE% observed in this study for TW80 is not clear but, it may be related to its ability to increase CA solubility in the aqueous core of the vesicles or near the interface of the novasomes. TW80 is a hydrophilic nonionic surfactant with a high HLB value of 15 and is known for its capacity to increase the aqueous solubility of lipophilic drugs [41,42].

The EE% decreased by changing the oleic acid:surfactant ratio from 1:1 to 1:2. This may be attributed to the increased permeability of the novasomes membrane due to the arrangement of added surfactant molecules within the lipid bilayer structure. This may have created pores within the membrane and increased its fluidity, eventually leading to decreased drug encapsulation [43].

As shown in Table 2, it was found that all the tested independent variables had a significant effect on EE%. The linear model was suggested to describe the EE% data. The Model F-value was 81.20, implying a significant model with a *p*-value < 0.01. Additionally, the data were well-fitted on the suggested linear model with *R*^2^ value of 0.9838. The difference between the predicted and adjusted *R*^2^ was less than 0.2, indicating best fitting by the linear model. The adequate precision was 24.7206, a value greater than 4, indicating an adequate signal and the applicability of the linear model to navigate the design space.

### 2.2. Influence of the Independent Variables on Novasomes PS (Y2)

**Figure 2:**
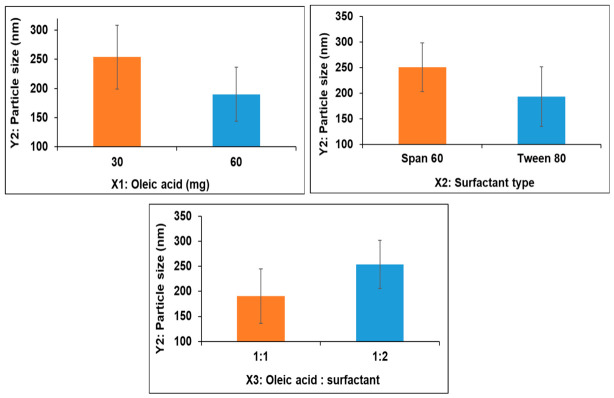
PS of the novasomes as a function of the tested independent variables.

The PS of CA novasomes ranged from 123.80 ± 1.44 nm to 313.95 ± 3.89 nm (Table 1). Figure 2 shows that it decreased by increasing the amount of oleic acid from 30 to 60 mg, probably due to the reduction in the surface free energy and formation of smaller vesicles [44]. Additionally, it may be related to oleic acid’s unsaturated structure, which enhances membrane fluidity and facilitates the formation of smaller vesicles [26].

TW80 vesicles had smaller PS compared to those of SP60 (Table 1, Figure 2). Some previous studies showed the opposite trend, where TW80 vesicles (HLB 15) had larger PS. This was attributed to the bulky head group and unsaturated structure of TW80, resulting in the formation of larger vesicles. In contrast, SP60 with a lower HLB (4.7) and saturated alkyl chain tends to form rigid, compact bilayer structures with a smaller particle size [45,46]. Nevertheless, other studies support the findings of our study, reporting that vesicles prepared with TW80 exhibit smaller PS than those prepared with SP60 under the same conditions. This effect was ascribed to the higher hydrophilicity and molecular structure of TW80, which promote the formation of small vesicles [47,48,49].

The ratio of oleic acid to surfactant plays an important role in determining the PS of vesicular systems such as novasomes. Table 1 and Figure 2 show that the PS increased upon changing the oleic acid:surfactant ratio from 1:1 to 1:2. This increase may be attributed to the increased amount of surfactant, which could expand the spacing within the vesicle bilayers. These findings are in agreement with previously reported results for comparable vesicular systems [26,50].

As represented in Table 2, all the tested independent variables had a significant effect on the PS. The suggested model for PS (Y2) was a linear one. The Model F-value was 34.85, implying a significant model with a *p*-value < 0.01. In addition, the data were well-fitted on the suggested linear model with a *R*^2^ value of 0.9631. The difference between the predicted and adjusted *R*^2^ was less than 0.2, which indicates best fitting by the linear model. The adequate precision, which measures the signal-to-noise ratio, was 17.6911. A signal–to–noise ratio value greater than 4 indicates an adequate signal and confirms the suitability of the linear model to navigate the design space [51].

### 2.3. Evaluation of the Vesicles PDI

PDI is a critical parameter in the characterization of vesicular drug delivery systems and other nanocarriers as it indicates the size distribution within a nanoparticle sample with values ranging from 0 (perfectly monodispersed sample) to 1 (highly heterogeneous). A PDI value ≤ 0.3 is acceptable for vesicular drug delivery systems, indicating a relatively uniform particle size distribution [52,53]. Homogeneity in size is required to ensure consistent drug release profiles, predictable pharmacokinetics, and consistent in vivo performance [52]. With the exception of formulations F4 and F8, the remaining formulations exhibited PDI values ranging from 0.3 to 0.4, indicating acceptable particle size distribution (Table 1). The formulation variables did not exhibit a consistent effect on PDI of the vesicles.

### 2.4. Influence of Independent Variables on the Novasomes ZP (Y3)

ZP is a measure of the surface charge of the vesicles. The higher the absolute value of ZP, the higher the physical stability of the colloidal dispersion. ZP values greater than +30 mV or lower than −30 mV result in strong electrostatic repulsion between the vesicles, preventing their aggregation and ensuring colloidal stability [54,55]. All the prepared novasomes exhibited negative ZP values ranging from −31.27 ± 1.04 mV to −39.70 ± 1.87 mV (Table 1). The negative surface charge may be due to the ionization of carboxylic acid groups of oleic acid. Oleic acid is a monounsaturated fatty acid having a carboxylic acid group (-COOH) that is ionized depending on the pH of the surrounding environment. The p*K*a of this -COOH is around 8.0, indicating that it has a significant degree of ionization at physiological pH [56]. Moreover, cholesterol has been documented to influence the negative surface charge of phospholipid membranes by altering the orientation and arrangement of lipid head groups [57].

**Figure 3:**
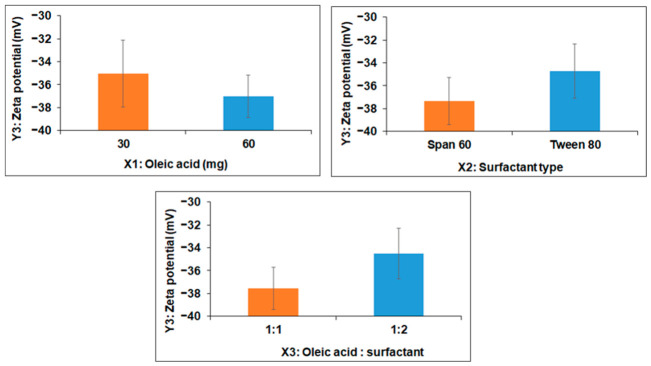
ZP of the novasomes as a function of the tested independent variables.

As shown in Figure 3, the negativity of the ZP increased as a result of increasing the concentration of oleic acid, which may be attributed to the increased presence of the free fatty acid bearing ionizable carboxyl groups.

The type of surfactant also affected the ZP values. Thus, the novasomes containing SP60 demonstrated more negative ZP values compared with those prepared with equal amounts of TW80. These findings may be attributed to the differences in their molecular structure and HLB values as reported in previous studies. SP60, with a low HLB (4.7), forms more rigid and closely packed bilayers that facilitate greater exposure of the vesicle surface leading to higher anion adsorption and surface charge accumulation. In contrast, TW80, which has a higher HLB value of 15 and bulky polyoxyethylene chains, creates steric hindrance that masks surface charges and results in less negative ZP [58,59].

Changing the oleic acid: surfactant ratio from 1:1 to 1:2 resulted in decreasing the absolute ZP values (Table 1). This observation is primarily attributed to the increased concentration of non–ionic surfactants, such as TW80 or SP60, which can shield or mask the negative charges of oleic acid carboxylate groups on the vesicle surface. The hydrophilic chains of these surfactants extend into the aqueous environment, creating a steric barrier that shields the effective surface charge. This, in turn reduces the magnitude of the ZP. Similar results were observed in previous studies where the negative values of ZP decreased by increasing the concentration of SP60 [60].

As shown in Table 2, all the tested independent variables had a significant effect on ZP. The suggested model of ZP (Y3) was a linear one. The Model F-value was 14.33 implying a significant model with a *p*-value <0.05. Additionally, the data were well-fitted on the suggested linear model with *R*^2^ value of 0.9149. The predicted and adjusted *R*^2^ were 0.6595 and 0.8510, respectively with a difference less than 0.2, indicating best fitting by the linear model. The adequate precision was 11.189, indicating an adequate signal and the suitability of the linear model for the navigation of the design space.

### 2.5. The Optimization Process

**Figure 4:**
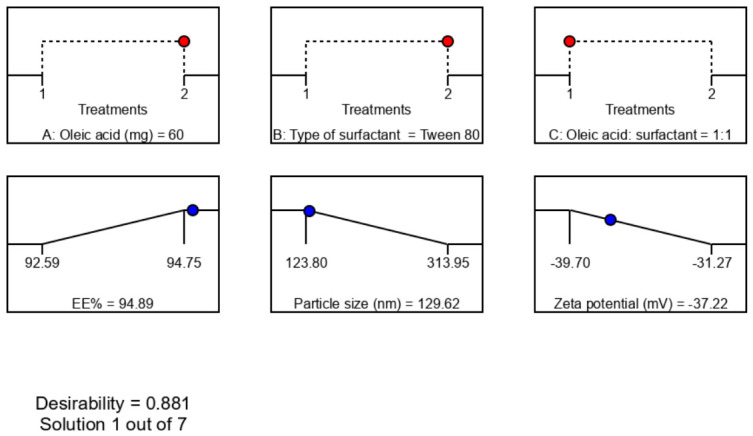
Properties of the optimized formulation of CA novasomes.

Numerical optimization was used to identify the optimal formulation by analyzing multiple factors, maximizing desirability, and predicting the best combination of variables. In this study, the goal was to obtain an optimized formulation with the highest EE%, the lowest PS, and the highest absolute ZP value. Figure 4 shows that the optimized formulation was prepared by a high level of X1 (60 mg oleic acid), high level of X2 (TW80), and low level of X3 (1:1). The predicted values of responses were 94.89% for EE% (Y1), 129.62 nm for PS (Y2), and −37.23 mV for ZP (Y3). The predicted values were close to the actual ones (formulation F6), supporting the validity of the optimization process (desirability index of 0.881). These favorable attributes of formulation F6 confirmed its suitability for further investigations.

### 2.6. TEM Measurements

**Figure 5:**
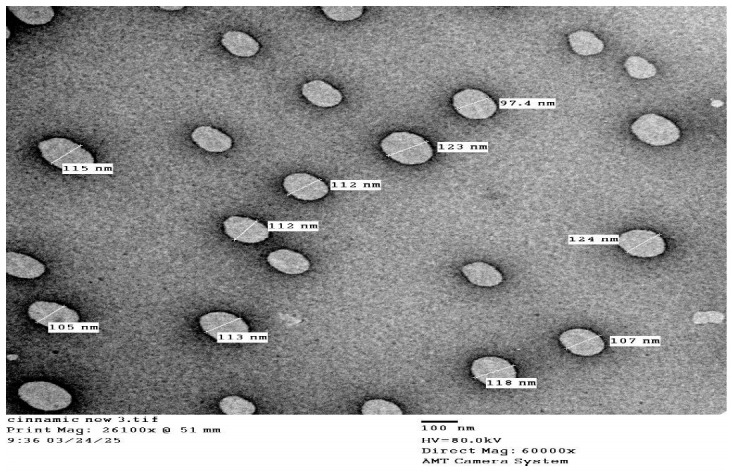
TEM photomicrograph of the optimized CA-loaded novasomes formulation.

Figure 5 shows a representative TEM photomicrograph of the optimized CA-loaded novasomes (formulation F6). The novasomes appear as spherical particles having a dark-colored wall and a light-colored interior, confirming their bilayer structure. They are also free from aggregation, which confirms their colloidal stability. The average size obtained from the TEM measurements was 112.6 ± 8.6 nm (*n* = 10). This size is slightly smaller than that obtained from DLS measurements (123.80 ± 1.44 nm, Table 1), which may be due to shrinkage of the vesicles because of dehydration during sample preparation for TEM measurements [61].

### 2.7. DSC Studies

**Figure 6:**
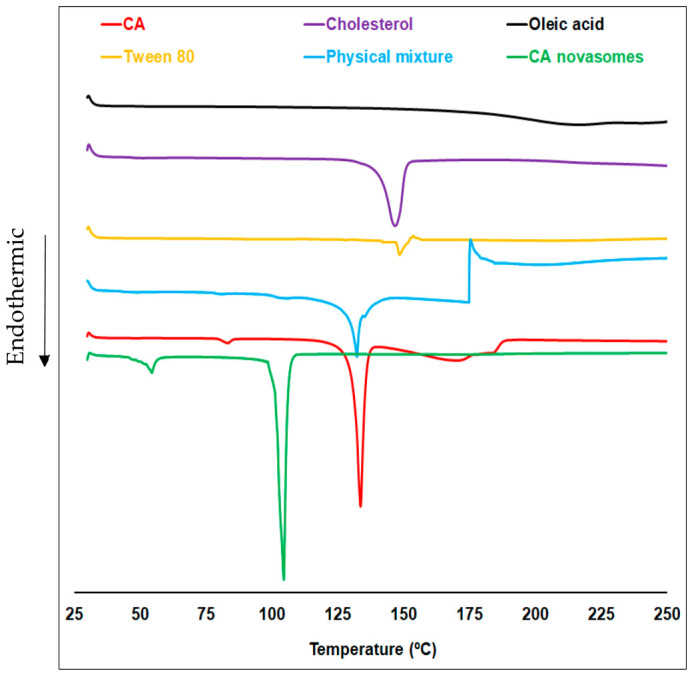
DSC thermogram of the optimized novasomes formulation (F6) in comparison with those of its individual components and their physical mixture.

CA thermogram shows a sharp endothermic peak at 133.7 °C, which is attributed to its melting endotherm (Figure 6) [62]. The thermogram of oleic acid shows no significant thermal events within the tested temperature range of 25–250 °C. Previous studies showed that oleic acid melting occurs at approximately 13 °C while its decomposition occurs at temperatures above 250 °C [63]. Cholesterol thermogram shows a sharp endothermic peak at 146.5 °C due to its melting [64]. The small endothermic peak at around 148 °C in TW80 thermogram is probably due to its flash point [65]. The thermogram of the physical mixture exhibits a sharp endothermic peak at around 132 °C, which is probably attributed to the drug melting endotherm. This melting peak had lower intensity compared with that of the drug alone, which is probably attributed to the dilution effect. The optimized novasomes formulation shows a sharp endothermic peak at around 104 °C, which is likely due to CA melting endotherm. The shift in the CA melting point from 133.7 °C to 104 °C upon encapsulation into the novasomes is likely due to several factors including reduction in drug crystallinity, nanometric size effects, and possible eutectic behavior within the vesicles. These effects could be taken as evidence of successful drug encapsulation within the novasomes and absence of chemical incompatibility. Similar effects have been previously observed for several other drugs [66,67].

### 2.8. FT–IR Studies

**Figure 7:**
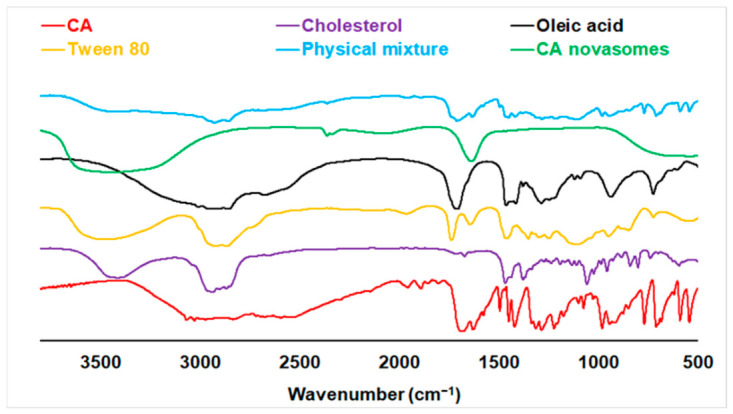
FT–IR spectra of the optimized novasomes formulation (F6) in comparison with those of its individual components and their physical mixture.

FT–IR studies were used to further evaluate possible interactions between CA and other components of the novasomes (Figure 7). CA spectrum shows a broad peak in the range of 2500 to 3300 cm^–1^ due to O-H group stretching. The peaks at 3026 and 2970 cm^–1^ are attributed to aromatic and aliphatic C-H stretching, respectively. The strong absorption peak at 1684 cm^–1^ is due to C=O stretching of the carboxylic acid groups [68]. Cholesterol spectrum shows a strong absorption band at 3406 cm^–1^ due to O-H group stretching. Additionally, the absorption bands at 2934, 2901 and 2866 cm^–1^ are ascribed to C-H stretching of asymmetric CH_3_, asymmetric CH_2_, and symmetric CH_2_, respectively [69]. The spectrum of oleic acid shows a small peak at around 3000 cm^–1^ due to C-H stretching of C=C–H. The band at 2924 cm^–1^ is attributed to -CH_2_ asymmetric stretching while the sharp peak at 1709 cm^–1^ is due to C=O stretching [70]. TW80 spectrum shows characteristic bands which appear at 3477, 2923, and 1735 cm^–1^ ascribed to O-H, C-H, and C=O groups stretching vibrations, respectively [71]. The physical mixture spectrum displays absorption bands at 2924, 2854, 1709, and 1632 cm^–1^, which are due to the absorption of its individual components. The spectrum of the optimized CA novasomes shows a broad absorption band centered at 3442 cm^−1^ and a distinct sharp band at 1634 cm^−1^. These bands are likely attributed to overlapping of the characteristic vibrations from the individual components of the formulation. No new peaks were observed in this spectrum, which rules out any covalent interactions between the drug and other novasomes components. However, the specific CA absorption bands disappeared in this spectrum, confirming successful encapsulation within the novasomes and absence of chemical interactions with other ingredients. This observation is consistent with earlier studies reporting disappearance of distinctive drug bands due to loading within the lipid bilayers [72,73].

### 2.9. Evaluation of CA Novasomal Gel

This evaluation was carried out to ensure the gel effectiveness, stability, and safety as a topical delivery system. As represented in Table 3, all the CA gel preparations were homogeneous, without any clumps, indicating uniform component distribution and appropriate consistency. Gel homogeneity is important to confirm that the system has no phase separation or aggregation, which confirms reproducible drug delivery performance.

#### 2.9.1. Spreadability Measurement

Spreadability is used as a measure of the ease with which a topical preparation spreads over the skin, which affects user comfort and dosing consistency. The spreadability values of the tested preparations followed this descending order: Free CA gel > plain carbopol gel > CA novasomal gel (Table 3). However, the differences were statistically significant only between free CA gel and CA novasomal gel (*p* < 0.05). This spreadability behavior is inversely related to the viscosity of the preparations (Table 4), as higher viscosity hinders the preparation spreadability due to increased thickening. Similar observations were previously reported for other vesicular gel preparations, which were explained on the basis of increased gel viscosity [74,75]. The increased gel viscosity upon incorporation of the novasomes into the carbopol gel was previously observed for other vesicular systems [76,77]. It was explained on the basis of formation of a colloidal network by the added vesicles, especially those containing cholesterol. Cholesterol molecules enhance the structural organization, making the gel more rigid and more resistant to flow under stress.

#### 2.9.2. pH and Drug Content of the Gels

The pH of the gel is an important parameter, which is used to ensure compatibility with the skin and minimize irritation. Previous studies showed that topical preparations with a pH in the range of 5–7 have high skin compatibility and minimum irritation [78]. Table 3 shows that the prepared gels had a pH in the range of 5.25 ± 0.08 to 5.65 ± 0.16, confirming their safety and lower irritation potential [79]. The drug content of the prepared gels was higher than 98% of the theoretical drug content (Table 3), which suggests minimal drug loss during preparation.

#### 2.9.3. Rheological Measurements

Rheological evaluation of gel preparations is important to optimize their physical properties, which directly affect their in vitro stability, drug release kinetics, and in vivo performance [80]. Table 4 shows that the plain carbopol gel had a viscosity of 2770 ± 35 cP at 49 rpm. The viscosity significantly decreased to 1583 ± 95 cP upon incorporation of 0.5% *w*/*w* drug for the free CA gel (*p* < 0.05). This reduction may be attributed to drug disruption of hydrogen bonding between carbopol chains and electrostatic interactions leading to decreasing structural integrity of the gel [81]. In contrast, incorporation of CA novasomes into the carbopol gel base did not result in a statistically significant change in viscosity (*p* > 0.05). This different behavior may be due to drug encapsulation within the vesicles, which limits its direct interaction with the carbopol chains. Viscosity values of the gels at low and high shear rates followed the same pattern as that measured at 49 rpm (Table 4).

**Figure 8:**
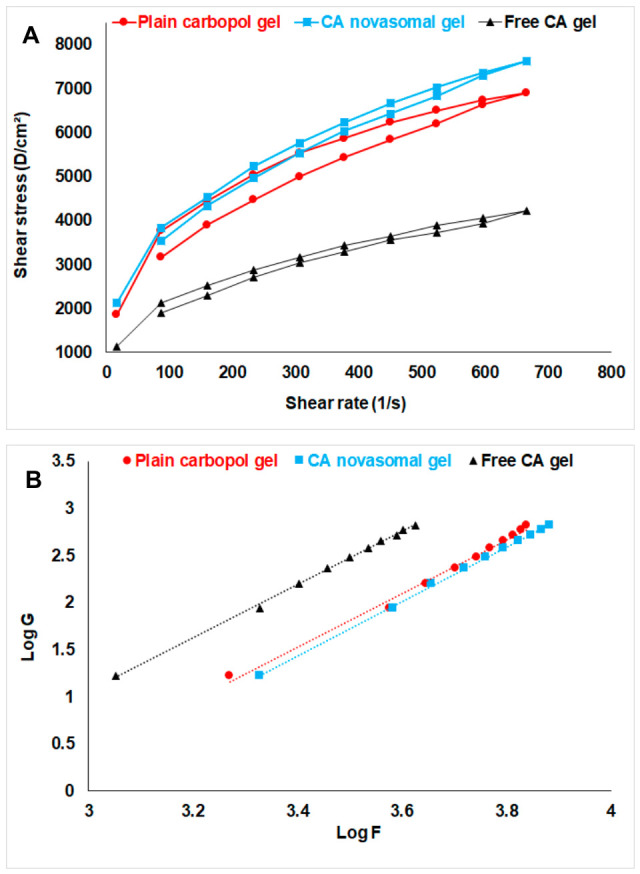
Rheological evaluation of CA novasomal gel in comparison with the plain carbopol gel and free CA gel at 25 °C. (**A**) Flow curve hysteresis and (**B**) Log shear rate (G) versus log shear stress (F).

To further characterize the rheological properties of the gel, the shear stress (D/cm^2^) was measured and plotted versus shear rate (s^–1^) (Figure 8A). A Bingham analysis was applied to obtain the yield stress, which is the stress above which a non–Newtonian material starts to flow (Table 4). Upon stress release, the material returns to its original shape [82]. The yield stress was highest for CA novasomal gel and lowest for free CA gel. The differences in the yield stress were statistically significant (*p* < 0.05). This trend is consistent with the measured viscosities of different gel preparations (Table 4). Previous studies have reported a positive relationship between the gel viscosity and yield stress. This is likely due to stronger polymeric network structures and a more extensive entanglement between the gel components at higher viscosity. Gel viscosity and yield stress determine its behavior under applied stress, which affects the gel properties such as stability, spreadability, and residence time at the site of administration [71,83]. The viscosity coefficient is another measure of the gel resistance to flow under applied stress. Similar to the yield stress, it also offers valuable insights into the internal structure of the gel and its behavior in response to shear forces. Table 4 shows that the viscosity coefficient had a trend consistent with that of both viscosity and yield stress.

Farrow’s constant (n) was calculated from the regression analysis of the logarithmic plot of shear rate versus shear stress and was used to further characterize the flow properties of the gels (Figure 8B). Values smaller than one indicate dilatant flow (shear-thickening systems) whereas values greater than one indicate pseudoplastic flow (shear-thinning systems) [84,85]. As shown in Table 4, the three tested gel preparations had Farrow’s constant values greater than one, confirming their shear-thinning behavior. Additionally, the flow index of the tested gels was less than one, which further confirms their shear–thinning behavior. This shear-thinning behavior of gels under applied stress is highly advantageous for topical drug delivery, as it reduces the effort required for uniform application and enhances patient compliance. Thus, shear-thinning gels have decreased viscosity under applied stress, such as spreading with the fingers, allowing convenient application. Upon removal of stress, they quickly recover their structural integrity and maintain adherence to the skin. This property ensures that the formulation is both patient–friendly and effective in maintaining the preparation at the application site [86]. The apparent viscosity (consistency index), which is derived from the power law model is a measure of the gel resistance to flow at low shear rates. Higher values indicate a structurally cohesive gel with greater viscosity at rest, which enhances drug retention and sustained release. On the other hand, lower values indicate better spreadability and reduced structural resistance [87,88]. Table 4 shows that the apparent viscosity followed this descending order: Plain carbopol gel > CA novasomal gel > free CA gel. The differences were statistically significant except between plain carbopol gel and CA novasomal gel.

Thixotropy measurements were performed to further characterize the effect of shear on the flow properties of the gels. Thixotropy indicates the decrease in viscosity with the applied shear stress, which ensures easy application and subsequent gradual recovery upon stress removal [86]. Previous studies have demonstrated that thixotropic behavior plays an important role in determining the performance of gel-based formulations, particularly in terms of stability, drug release kinetics, and patient compliance [89]. All the tested gels exhibited thixotropic behavior as shown by their ability to regain their viscosity upon shear removal (Figure 8A). Table 4 demonstrates that the plain carbopol gel had the highest thixotropy followed by CA novasomal gel and finally free CA gel. The differences were statistically significant except between free CA gel and CA novasomal gel.

### 2.10. In Vitro CA Release Studies

**Figure 9:**
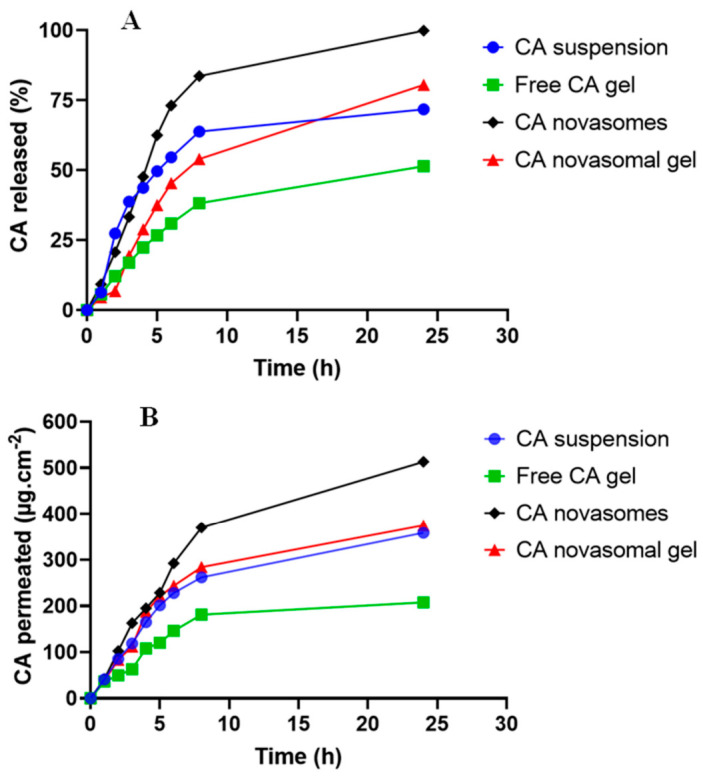
(**A**) In vitro release profiles of CA from various formulations. (**B**) Ex vivo skin permeation of various CA formulations. The experiments were carried out in pH 7.4 phosphate buffered saline at 37 ± 0.5 °C.

Figure 9A shows the in vitro release profile of CA novasomal gel in comparison with the aqueous CA novasomal dispersion, aqueous CA suspension and free CA gel. The experiment was designed to evaluate the influence of two factors on drug release: CA encapsulation within the novasomes and the incorporation of novasomes into a carbopol gel base. Up to 4 h, the release profiles were almost the same for the four tested preparations. After that, it was evident that CA encapsulation within the novasomes led to a significant increase in the percent drug released (*p* < 0.05). For instance, the percent CA released was 49.60 ± 0.64% for CA suspension and 62.51 ± 0.76% for CA novasomes after 5 h. This trend persisted throughout the 24-h release period, with the difference in drug release percentages increasing over time. After 24 h, 71.76 ± 0.79% and 99.84 ± 1.38% were released for CA suspension and CA novasomes, respectively. A similar effect was also observed for the two gel samples, i.e., CA novasomal gel had significantly higher percent drug released compared with the free CA gel (*p* < 0.05). The higher CA release rate from the novasomal preparations may be due to the smaller particle size of the vesicles compared with the coarse particles of CA suspension [90]. Reduced drug particle size increases the specific surface area available for dissolution, which enhances the drug release rate [91]. Moreover, the novasomes contain TW80, which was previously shown to improve the aqueous solubility of poorly soluble drugs through micellar solubilization [41,42].

Regarding the effect of incorporation into the carbopol gel, a general trend of reduced drug release rate was observed for the gel formulations compared to the drug suspension and drug–loaded novasomes. Thus, the percent CA released after 5 h was 26.69 ± 1.03% for the free CA gel compared with 49.60 ± 0.64% for CA suspension. Additionally, CA novasomal gel had a percent release of 37.53 ± 0.78% compared with 62.51 ± 0.76% for CA novasomes after 5 h. By the end of the 24–h experiment period, the percent CA released followed this order: CA novasomes > CA novasomal gel > CA suspension > free CA gel. The differences between these formulations were statistically significant (*p <* 0.05). Similar effects have been reported in previous studies, which attributed them to the high viscosity of the gel that limited drug diffusion through the gel matrix [81,92].

### 2.11. Ex Vivo Skin Permeation Study

The skin permeation profiles of CA from different formulations through rat abdominal skin are illustrated in Figure 9B. Excised rat skin was used due to its availability, cost-effectiveness, and structural similarity to human skin [93]. Additionally, human skin has limited availability, high cost, and strict ethical constraints [94]. However, excised rat skin was previously shown to have higher permeability than human skin, suggesting that permeability data should be interpreted carefully when extrapolated to human use [95]. All the tested formulations demonstrated biphasic permeation profiles, characterized by rapid permeation during the first 8 h followed by a slower permeation rate for the remainder of the study. After 24 h, the cumulative permeated CA amount ranked as follow with statistically significant differences: CA novasomes > CA novasomal gel > CA suspension > free CA gel. CA suspension produced relatively slow permeation rate with a steady state flux of 34.19 ± 0.54 µg/cm^2^ and cumulative CA permeation after 24 h of 60.91 ± 0.31% (Table 5). This is likely attributed to the CA hydrophobic nature, which limits its dissolution in the aqueous medium, resulting in slow permeation. This observation is consistent with previous results, which showed limited skin permeation of hydrophobic drugs [96]. In contrast, CA novasomes produced around 1.4–fold higher CA flux relative to CA suspension after 24 h with cumulative percent CA permeated of 87.25 ± 0.41% (Table 5). This enhancement is probably attributed to several factors including the nanometric PS of the vesicles (123.80 ± 1.44 nm, Table 1). This nanometric PS enhances the vesicles ability to permeate through various skin layers, which was also observed in other studies [97]. Additionally, the presence of TW80 in the vesicles may have increased their fluidity, which is another factor known to boost drug skin permeation [98]. The novasomes also contained oleic acid, which was previously shown to reversibly enhance the fluidity of the stratum corneum and act as a permeation enhancer leading to improved drug permeability [99]. Another observation was that the gel formulations, including free CA gel and CA novasomal gel, exhibited markedly slower permeation parameters compared with the corresponding liquid preparations (Table 5). Thus, free CA gel had 1.73-fold lower percent cumulative CA permeated after 24 h relative to the aqueous CA suspension. Additionally, CA novasomal gel had around 1.4-fold lower percent cumulative CA permeated after 24 h relative to the CA novasomes (Table 5). This slower permeation from the gels is widely reported in the literature and is usually attributed to the increased viscosity of the gel, as well as the diffusional barrier imposed by the gel three-dimensional network, which limits the drug release from the preparation [75,100]. It is also noteworthy that the permeation profiles are consistent with those of the in vitro drug release observed in Figure 9A.

### 2.12. CA Deposition in Various Skin Layers

**Figure 10:**
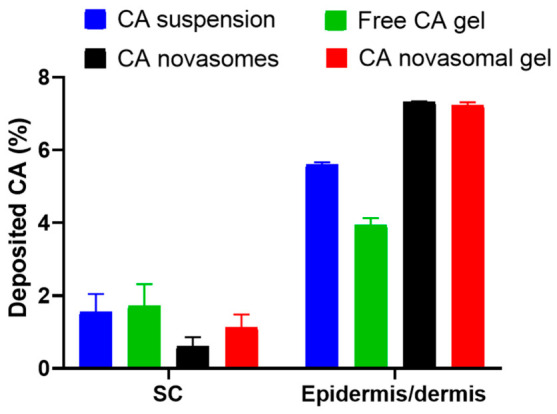
Percentage of CA deposited in stratum corneum (SC) and epidermis/dermis following topical application of different CA preparations.

The skin deposition results after 24 h demonstrate formulation–dependent differences in CA amounts deposited in both the stratum corneum (SC) and viable epidermis/dermis layers (Figure 10). Free CA gel exhibited the highest retention in the SC layer, followed by CA suspension, while both CA novasomes and its gel formulation showed the lowest deposition values. However, the differences between all formulations were not significant. In contrast, the deposition in the epidermis/dermis skin layers was as follow: CA novasomes > CA novasomal gel > CA suspension > free CA gel. The differences were statistically significant except that between CA suspension and CA novasomal gel. It is noteworthy that this order is consistent with the CA permeation results shown in Figure 9B. These findings confirm the ability of CA–loaded novasomes to enhance drug concentration into deep skin layers while reducing excessive retention within the SC. Deformable phospholipid vesicles such as novasomes have shown the ability to interact with and disrupt the SC lipid matrix, specifically by modifying the intracellular lipid lamellae structure. This increases the SC lipid fluidity and enables drug delivery to the viable epidermis and dermis [26,101,102]. Moreover, the drug vesicles may also act as a drug reservoir that modulate the drug release at or near the skin surface, enabling facilitated drug diffusion through the SC via the increased concentration gradient effect [101]. This deeper skin delivery of CA is particularly important for bacterial skin infections. Pathogens such as *S. aureus* and other microorganisms can colonize the viable epidermis, hair follicles, and other dermal structures rather than remaining confined superficially on the SC. Achieving high drug concentrations in deep skin layers is essential for improving the drug local bioavailability and for efficient eradication of the skin bacterial infections [7,103,104,105].

### 2.13. In Vitro Antibacterial Studies

#### 2.13.1. Screening of Antibacterial Activity

**Figure 11:**
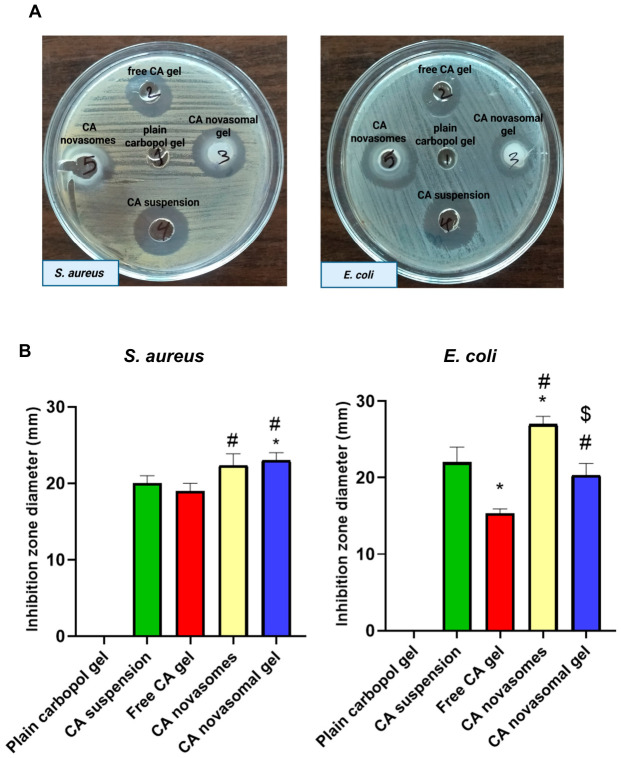
Antibacterial activity of various CA formulations against *S. aureus* and *E. coli*. (**A**) photomicrographs of the agar plates (1; plain carbopol gel, 2; free CA gel; 3; CA novasomal gel, 4; CA suspension, 5; CA novasomes). (**B**) Inhibition zone diameters (mm). * significant compared with CA suspension at *p* < 0.05, # significant compared with free CA gel at *p* < 0.05. $ significant compared with CA novasomes at *p* < 0.05. Statistical analysis was done using one–way analysis of variance (ANOVA) with Tukey post–hoc test.

The agar gel diffusion method was used to evaluate the potential of various CA formulations to improve CA antibacterial activity against *S. aureus* and *E. coli*, as representatives of Gram-positive and Gram-negative bacteria, respectively. The plain carbopol gel exhibited no antibacterial activity against both strains (Table 6 and Figure 11). In contrast, all CA-containing formulations demonstrated varying degrees of antibacterial effect. Two general effects were noticed for the CA formulations: the drug-loaded novasomes exhibited higher inhibition zones than the drug suspension, and the gel formulations showed smaller zones than their corresponding formulations. For *S. aureus*, the differences in the inhibition zones were significant (*p* < 0.05) except between CA suspension and free CA gel, CA suspension and CA novasomes, and CA novasomes and CA novasomal gel. For *E. coli*, all the differences were significant (*p* < 0.05) except between CA suspension and CA novasomal gel and between free CA gel and CA novasomal gel. The minimum inhibitory concentration (MIC) was identical for both CA suspension and CA novasomes (0.312 mg/mL) against *S. aureus* (Table 6). In contrast, CA encapsulation into the novasomes resulted in a two–fold reduction in its MIC against *E. coli*.

#### 2.13.2. Anti–Biofilm Assay

The anti–biofilm activity of CA suspension and CA novasomes was assessed against *S. aureus* and *E. coli*. The novasomes-loaded drug exhibited greater biofilm inhibition compared to the drug suspension. Thus, the novasomes achieved a 90.0 ± 0.1% inhibition rate against *S. aureus* compared with 85.6 ± 0.3% for CA suspension. Similarly, the novasomes showed a 92.7 ± 0.5% inhibition rate against *E. coli* compared with 81.3 ± 0.1% for CA suspension.

Collectively, the results of these in vitro antibacterial studies confirmed the enhancement of CA antibacterial effects upon encapsulation within the novasomes. This enhancement may be attributed to improved uptake of the novasomes-loaded drug by bacterial cells, as well as enhanced drug release from the vesicles [26]. Specifically, the nanometric size of the vesicles may have facilitated closer interaction with bacterial cells, resulting in increased intracellular drug concentration and enhanced bactericidal activity [106,107]. The nanosized vesicles may also penetrate more efficiently through the extracellular polymeric substance (EPS) of bacterial biofilms, thereby improving drug access to embedded bacterial communities and enhancing biofilm eradication [108,109]. In addition, the in vitro release studies demonstrated enhanced CA release from the novasomes compared with the free drug suspension, which may have increased drug availability at the site of bacterial growth and contributed to improved antibacterial efficacy [25,29].

The enhanced anti–biofilm activity may also be related to the intrinsic ability of cinnamic acid to interfere with bacterial quorum-sensing systems, which play a central role in biofilm formation, maturation, and virulence regulation. Prolonged exposure of bacterial cells to CA through the sustained release behavior of the novasomal carrier may therefore enhance its quorum-sensing inhibitory effects and contribute to the observed reduction in biofilm formation [110,111,112]. Consequently, the superior anti-biofilm activity of the optimized novasomes is likely the result of a combination of nanoscale size, improved drug availability, and enhanced penetration into biofilm matrices. Previous studies showed comparable results where ciprofloxacin-loaded niosomes had lower MIC and higher anti-biofilm effects than the free drug [113]. Similarly, noisome-loaded quercetin achieved higher antibacterial and anti-biofilm activities compared with the free drug [114].

## 3. Materials and Methods

### 3.1. Materials

CA was purchased from Loba Chemi Pvt. Ltd., (Mumbai, India). Tween 80 (TW80) was obtained from Sigma Aldrich Co. (St. Louis, MO, USA). Cholesterol, oleic acid, and Span 60 (SP60) were purchased from Central Drug House Ltd., (New Delhi, India). Mueller-Hinton agar, resazurin, nutrient broth, brain heart infusion broth, and Sabouraud dextrose agar (SDA) were purchased from Thermo Fisher Scientific (Waltham, MA, USA).

### 3.2. Design of CA Novasomes According to a 2^3^ Factorial Design

A 2^3^ factorial design was applied using Design–Expert software (Design–Expert 11 software, Stat–Ease, Minneapolis, MN, USA). Three independent variables were tested, each at two levels. These variables were oleic acid amount (X1), type of surfactant (X2), and oleic acid: surfactant ratio (X3) (Table 7). The dependent variables (responses) were drug entrapment efficiency (EE%, Y1), particle size (PS, Y2), and zeta potential (ZP, Y3).

### 3.3. Preparation of CA–Loaded Novasomes

CA–loaded novasomes were prepared using the previously reported ethanol injection method with slight modifications [115]. Briefly, the designated amounts of CA, oleic acid, surfactant (TW80 or SP60), and cholesterol were dissolved in ethanol at 60 °C in a small beaker (Table 1). The ethanolic solution was slowly added to a beaker containing water heated at the same temperature and stirred at 700 rpm on a magnetic stirrer (Daihan Labtech Co., Ltd., Namyangju, Korea). The appearance of turbidity indicated the successful novasomes formation. The preparations were kept on the stirrer for 2 h until complete evaporation of ethanol. All preparations were subjected to sonication in a water bath sonicator for 30 min for reduction of the vesicle size [26]. They were then kept at 4°C for further characterization.

### 3.4. Evaluation of EE%

The EE% was measured by an indirect method involving centrifuging an aliquot (2 mL) of each formulation using a cooling centrifuge at 20,000 rpm for 2 h [116]. The clear supernatant was separated and analyzed for the unentrapped drug, after appropriate dilution, using a UV spectrophotometer (Cary 5000 UV–Vis Spectrophotometer, Santa Clara, CA, USA) at a λ_max_ of 272 nm. The EE% was calculated using the following equation [61]:

(1) $$EE\%=\frac{Total\;amount\;of\;CA-Unentrapped\;amount\;of\;CA}{Total\;amount\;of\;CA}\times 100$$

### 3.5. Evaluation of PS, PDI, and ZP

These parameters were determined at room temperature by the dynamic light scattering technique using a Malvern ZetaSizer Nano Series ZS 90 (Malvern Instruments, Malvern, UK). Aliquots of the preparations were diluted (1:200 *v*/*v*) with distilled water before measurements. All samples were measured in triplicate at an angle of 173° [117].

### 3.6. Selection of the Optimized Formulation

The optimum formulation of CA-loaded novasomes was selected based on the highest EE%, smallest PS, and highest ZP.

### 3.7. Transmission Electron Microscopy (TEM) Measurements

A TEM machine (JEOL^®^, Tokyo, Japan) was used to evaluate the surface characteristics and size of the optimized formulation of CA novasomes. A drop of the optimized formulation (formulation F6) was applied to a collodion-coated copper grid after being diluted with distilled water (1:10 *v*/*v*). After staining with uranyl acetate solution (2% *w*/*v*), the sample was allowed to dry at room temperature and imaged by the TEM machine operating at 80 kV [35]. Size from this measurement was compared to that obtained from the dynamic light scattering technique.

### 3.8. Differential Scanning Calorimetry (DSC) Studies

DSC thermograms of the optimized F6 formulation, its individual ingredients (CA, cholesterol, oleic acid, and TW80) and their physical mixture were obtained using a TA Discovery DSC 25 (TA Instruments, Waters LLC, New Castle, DE, USA). Samples (2–5 mg) were accurately weighed and tightly sealed in aluminum pans. The sealed pans were heated at a rate of 10°C/min in the 25–250 °C range under nitrogen gas flowing at 25 mL/min.

### 3.9. Fourier–Transform Infrared (FT–IR) Studies

The FT-IR spectra of the optimized F6 formulation, its individual ingredients (CA, cholesterol, oleic acid, and TW80) and their physical mixture were recorded using a Thermo Scientific™ Nicolet™ iS10 FT-IR spectrometer (Thermo Fisher Scientific, Waltham, MA, USA). The spectra were obtained in the range of 4000 to 400 cm^–1^ at room temperature with a resolution of 4 cm^–1^.

### 3.10. Preparation of CA Novasomal Gel

Plain carbopol 934 gel was prepared by dispersing 1.0 g of the polymer in 100 mL of distilled water. The dispersion was stirred on a magnetic stirrer and drops of methylamine solution in ethanol were added to neutralize the polymer and form the gel. Free CA gel was prepared by dispersing carbopol into an aqueous suspension of CA, followed by neutralization and gelation using methylamine. CA novasomal gel was prepared by dispersing carbopol into the optimized formulation dispersion (formulation F6) while stirring using a magnetic stirrer at 500 rpm. The resulting mixture was neutralized with methylamine to induce gel formation. All formulations were gently stirred to ensure uniform consistency and stored in sealed containers for further evaluation. The final carbopol concentration was 1.0% *w*/*w* in all preparations. CA concentration was 0.5% *w*/*w* in free CA gel and CA novasomal gel.

### 3.11. Evaluation of Gel Formulations

#### 3.11.1. Evaluation of Homogeneity

The homogeneity of the gels was visually inspected to ensure uniformity and absence of lumps. A small amount of the gel was applied between the thumb and index finger to check for any lumps, or clumps. A well-formulated gel should have a smooth, even texture without particulate matters [118].

#### 3.11.2. Measurement of Spreadability

Half gram of the tested formulation was placed on a premarked circle on one glass slide and another slide was pressed on the first one using a standard load of 500 g for 1 min. Subsequently, the diameter of the gel spread was measured and taken as the spreadability value [119].

#### 3.11.3. pH Measurement

An Adwa pH meter (AD8000, Adwa, Szeged, Hungary) was utilized to measure the pH of the prepared gels. The pH meter was calibrated before each measurement using solutions buffered at pH 4, 7 and 10.

#### 3.11.4. Rheological Evaluation

An AMETEK Brookfield Cap 2000+ viscometer (Middleborough, MA, USA) with spindle number 5 was used to characterize the rheograms of the tested gel samples at 25 ± 0.1 °C. The shear rate was varied between 86.66 and 666.7 s^–1^, equivalent to a speed of 27–201 rpm. The shear stress was recorded and plotted as a function of the shear rate. The samples were kept for one min at rest to avoid any aging effect [120]. The yield stress and flow index were calculated from the rheograms using the Bingham and Power Law analysis plots, respectively available in the Capcalc V 3.0 build 20-1 software (AMETEK Brookfield, Middleborough, MA, USA). The percent confidence of fit ranged from 94.2 ± 0.05% to 95 ± 0.7%.

The thixotropy measurements were made over a shear rate of 17–667 s^–1^, equivalent to a speed of 5 to 200 rpm. The speed started at 5 rpm and gradually increased at a constant rate up to 200 rpm, with time intervals of 20 s between each increment. The speed was then reduced gradually using the same order as the increasing speed until reaching the starting speed [121]. The shear stress (dyne/cm^2^) was plotted versus the shear rate (s^–1^) and the areas under the upward and downward curves were calculated using the trapezoidal rule. The area difference was taken as the hysteresis loop area [122]. The logarithm of shear rate was plotted versus the logarithm of shear stress from the upward flow curve using the following Farrow’s equation [121]:

Log G = n log F − log η′(2)

where G is the shear rate (s^−1^), F is shear stress (dyne/cm^2^), η′ is the viscosity coefficient, and n is the Farrow’s constant [122]. Other flow parameters were calculated using the Power Law analysis plots available in the Capcalc V 3.0 build 20–1 software from the following equation:

Ʈ = ƞ Ƴ^n^ (3)

where Ʈ is the shear stress (dyne/cm^2^), ƞ is the consistency index (apparent viscosity), Ƴ is the shear rate (s^−1^), and n is the flow index.

#### 3.11.5. Measurement of Drug Content

A known amount of the tested gel was dissolved in ethanol, and the resulting solution was analyzed spectrophotometrically at a λ_max_ of 272 nm. The absorbance was converted to drug concentration using a calibration curve and the percent CA content of the gel was calculated.

### 3.12. Drug Release Studies

A Franz diffusion cell was used to evaluate the in vitro release of CA from its novasomal gel in comparison with CA suspension, CA novasomes, and free CA gel. An accurate amount of each formulation (equivalent to 1 mg drug) was added to the donor compartment, which was separated from the receptor compartment by a cellophane membrane having a diameter of 1.7 cm. As a release medium, 12.5 mL of phosphate–buffered saline (PBS) pH 7.4 was added to the receptor compartment. The release media were kept at 37 °C and magnetically stirred at 100 rpm during the experiment. Samples (3 mL) were withdrawn at different time intervals (1, 2, 3, 4, 5, 6, 8, and 24 h) and the concentration of CA was measured using a UV-Vis spectrophotometer at a λ_max_ of 272 nm.

### 3.13. Skin Permeation Study

The permeation of CA suspension, free CA gel, CA novasomes, and CA novasomal gel was evaluated through rat abdominal skin employing the Franz diffusion cell apparatus. The study protocol was approved by the research ethics committee of Sinai University (Approval No. SU.REC.2025 (61 H) date of approval: 7 July 2025). The study adhered to internationally accepted standards for animal research, following the 3Rs principle. These experiments followed the ARRIVE guidelines and the National Research Council Guide for the Care and Use of Laboratory Animals, 8th edition (National Academies Press, Washington, DC). Abdominal skin of Sprague–Dawley male rats (*n*= 4, 250–300 g) was obtained following their sacrifice. Uniform pieces of the skin with no irregularities or crevices (three pieces for each formulation) were placed between the donor and receptor chambers of the cell, making sure that the stratum corneum was facing the donor chamber. A sample from each formulation (equivalent to 1 mg CA) was placed in the donor chamber. The receptor chamber was filled with 20 mL of pH 7.4 PBS maintained at 37 ± 0.5 °C and stirred at 100 rpm. At specified time intervals (1, 2, 3, 4, 5, 6, 8, and 24 h), 2-mL samples were withdrawn from the receptor chamber and replaced with an equal volume of fresh medium kept at the same temperature. CA concentration in these samples was determined spectrophotometrically at 272 nm.

To determine CA deposition in various skin layers, the skin samples were carefully removed from the diffusion apparatus at the end of the permeation study. They were then washed with distilled water to remove the tested formulations residues. The skin samples were fixed with small pins on cork plates. The stratum corneum was removed by the tape–stripping technique by the aid of an adhesive tape. To remove the horny layer, ten consecutive strips were applied to each skin piece, making sure uniform pressure was applied. To avoid interference from the formulation residues, the first strip was discarded. Following the removal of the stratum corneum, the remaining skin was cut into small pieces. To extract CA from the collected tapes and skin pieces, they were placed separately overnight in methanol. The methanol mixtures were vortexed for 5 min followed by 5–min sonication to facilitate full CA extraction. The CA concentration of the samples was quantified spectrophotometerically at 272 nm. The permeated CA amount per unit surface area (µg/cm^2^) was plotted against time (h) to construct the permeability curves. The CA steady state flux (*J*_ss_, µg/cm^2^.h) was obtained from the linear regression line slope. The apparent permeability coefficient (Papp, cm/h) was calculated from Equation (4).

(4) $$P_{app}=\frac{J_{ss}}{C_{0}}$$

where *J*_ss_ is the steady state flux and C_0_ is the CA initial concentration.

The percent enhancement factor (EF%) was determined from Equation (5).

(5) $$EF\left(\%\right)=\frac{J_{ss\left(A\right)}\times 100}{J_{ss\left(B\right)}}$$

*J*_ss(A)_ and *J*_ss(B)_ are the flux of test preparation and CA suspension, respectively.

### 3.14. In Vitro Antibacterial Studies

#### 3.14.1. Screening of Antibacterial Activity

The agar cup diffusion technique was employed to evaluate the antibacterial efficacy of CA suspension, free CA gel, CA novasomes and CA novasomal gel against *Staphylococcus aureus* (ATCC 6538) and *Escherichia coli* (ATCC 8739). The bacterial strains were revived by overnight incubation at 37 °C on Mueller–Hinton agar plates. A freshly prepared bacterial suspension was then adjusted to a turbidity value equivalent to 0.5 McFarland standard and evenly spread over the surface of the agar plates using sterile swabs. Wells measuring 1 cm in diameter were created in the agar using a cork borer, and the resulting agar plugs were carefully removed. An aliquot of 100 μL of each tested formulation was placed into the wells. The plates were incubated overnight at 37 °C, and the diameters of the inhibition zones formed around the wells were measured [123,124]. CA concentration in all samples was 0.5% *w*/*w*.

#### 3.14.2. Determination of Minimum Inhibitory Concentration (MIC)

MIC determination was carried out following previously described procedures with some modifications using the micro-dilution assay method in a 96-well microtiter plate [123,124]. Briefly, nutrient broth (100 μL) was added to each well of the microtiter plate. Next, 100 μL of the tested formulations and positive antibacterial control (gentamicin 0.025 mg/mL) were added to the wells of the first vertical row. Mixing was carried out followed by two–fold serial dilution and discarding 100 μL from the last well. Diluted *S. aureus* and *E. coli* suspensions (100 μL, 1.5 × 10^5^ CFU/mL) were added to each well. The plates were incubated overnight at 37 °C and 10 μL of resazurin dye was added to each well and incubated for further 4 h at 37 °C to interpret the MIC values.

#### 3.14.3. Inhibition of Bacterial Biofilm Formation

The anti–biofilm potential of various CA formulations was tested against *S. aureus* and *E. coli* following a previously described protocol with minor modifications [125]. The bacterial strains were cultured in brain heart infusion broth and incubated overnight at 37 °C. The tested formulations at concentrations lower than MIC were transferred to 96-well microtiter plates and mixed with diluted bacterial cultures (1.5 × 10^8^ CFU/mL). Wells containing only broth were used as negative controls, while wells containing untreated bacterial cultures and broth served as positive controls. The plates were incubated at 37 °C for 24 h and the contents of each well were gently removed. To remove non-adherent bacteria, the wells were washed 2–5 times with 200 μL of sterile saline. The formed bacterial biofilms were dried at 65 °C for 30 min and stained with 200 μL of 0.1% crystal violet solution. Each well was washed five times with normal saline (200 μL) to remove excess dye. Stained biofilms were dried at ambient temperature and solubilized using 33% glacial acetic acid. The absorbance was measured at 570 nm using a SPECTROstar Nano microtiter plate reader (BMG Labtech, Ortenberg, Germany) after 15–30 min. Anti-biofilm effect was calculated by comparing the treated wells absorbance to that of the untreated positive controls. The percentage of biofilm inhibition was calculated using the following equation:

(6) $$Percentage\;inhibition=\frac{Absorbance\;of\;positive\;control-Absorbance\;of\;treated\;well}{Absorbance\;of\;positive\;control}\times 100$$

A good inhibition was indicated by a percentage greater than 50% while a percentage of 0–50% indicated weak inhibition. A negative value (below 0%) was considered as biofilm enhancement [125,126].

### 3.15. Statistical Analysis

Each experiment was repeated three times at least and the results were shown as mean ± SD. Statistical analysis was performed using the one-way analysis of variance (ANOVA) with Tukey post-hoc test available in GraphPad Prism software version 8.0.1 (GraphPad Software Inc., La Jolla, CA, USA). Normality was assessed using the Shapiro-Wilk test in GraphPad Prism. Differences were considered statistically significant for *p*-value of less than 0.05.

## 4. Conclusions

CA-loaded novasomes were successfully prepared and optimized using a 2^3^ factorial design employing oleic acid amount, type of surfactant, and oleic acid: surfactant ratio as independent variables. The independent variable exhibited significant effects on the measured responses. Strong correlation was observed between the expected and measured outcomes, which confirms the robustness, reliability, and predictive capability of the suggested model. The optimized formulation showed favorable properties, including high EE%, nanometric PS, small PDI, and high absolute ZP value. When incorporated into carbopol gel, this formulation exhibited shear-thinning behavior, skin-compatible pH, and acceptable spreadability, supporting its potential for clinical application. The novasomes enhanced CA in vitro release rate and permeation through rat abdominal skin compared with the drug suspension. It also improved drug deposition within the viable epidermis/dermis skin layers, which confirms the ability of the developed formulation to reach and eradicate deep skin infection. The in vitro antibacterial studies confirmed the ability of CA novasomal formulations to improve its effects against *S. aureus* and *E. coli*, as indicated by higher inhibition zones, lower MICs, and higher anti-biofilm effects. The obtained results demonstrate that encapsulation of CA within novasomes enhances its topical skin delivery and antibacterial activity, thereby highlighting their potential to overcome its inherent limitations and improve its therapeutic efficacy. The limitations of the study include the absence of physical and chemical stability assessments of the optimized formulation, as well as the evaluation of its skin safety and irritation potential. These aspects warrant further investigation and will be addressed in future studies.

## Figures and Tables

**Table 1:** Composition and properties of the prepared CA novasomes.

Formulation	Oleic Acid (X1, mg)	Surfactant (X2)	Oleic Acid:Surfactant (X3)	EE% (Y1)	PS (Y2, nm)	ZP (Y3, mV)	PDI
F1	30	SP60	1:1	92.90 ± 0.14	254.87 ± 16.61	−38.33 ± 0.83	0.41 ± 0.03
F2	30	TW80	1:1	94.22 ± 0.04	181.33 ± 4.63	−35.53 ± 1.36	0.26 ± 0.02
F3	30	SP60	1:2	92.59 ± 0.25	313.95 ± 3.89	−35.10 ± 1.87	0.42 ± 0.04
F4	30	TW80	1:2	93.86 ± 0.30	265.43 ± 42.27	−31.27 ± 1.04	0.57 ± 0.02
F5	60	SP60	1:1	93.89 ± 0.08	201.57 ± 3.31	−39.70 ± 1.87	0.43 ± 0.01
F6	60	TW80	1:1	94.75 ± 0.05	123.80 ± 1.44	−36.63 ± 0.61	0.38 ± 0.01
F7	60	SP60	1:2	93.45 ± 0.16	232.80 ± 2.20	−36.23 ± 0.06	0.38 ± 0.04
F8	60	TW80	1:2	94.54 ± 0.25	201.93 ± 19.43	−36.50 ± 1.56	0.66 ± 0.19

**Table 2:** ANOVA analysis of the independent variables’ effect on the measured responses.

Y1: EE%						
Source	Sum of Squares	df	Mean Square	F-Value	*p*-Value	
Model	3.96	3	1.32	81.20	0.0005	Significant
X1: Oleic acid amount	1.17	1	1.17	71.92	0.0011	
X2: Type of surfactant	2.58	1	2.58	158.31	0.0002	
X3: Oleic acid: surfactant	0.2178	1	0.2178	13.38	0.0216	
Residual	0.0651	4	0.0163			
Cor Total	4.03	7				
*R* ^2^	Predicted *R*^2^		Adjusted *R*^2^		Adeq Precision	
0.9838	0.9354		0.9717		24.7206	
Y2: PS						
Source	Sum of Squares	df	Mean Square	F-Value	*p*-Value	
Model	22,783.52	3	7594.51	34.85	0.0025	Significant
X1: Oleic acid amount	8158.99	1	8158.99	37.44	0.0036	
X2: Type of surfactant	6651.87	1	6651.87	30.52	0.0052	
X3: Oleic acid: surfactant	7972.67	1	7972.67	36.58	0.0038	
Residual	871.80	4	217.95			
Cor Total	23,655.33	7				
*R* ^2^	Predicted *R*^2^		Adjusted *R*^2^		Adeq Precision	
0.9631	0.8526		0.9355		17.6911	
Y3: ZP						
Source	Sum of Squares	df	Mean Square	F-Value	*p*-Value	
Model	39.58	3	13.19	14.33	0.0132	Significant
X1: Oleic acid amount	7.67	1	7.67	8.33	0.0447	
X2: Type of surfactant	13.61	1	13.61	14.78	0.0184	
X3: Oleic acid: surfactant	18.30	1	18.30	19.88	0.0112	
Residual	3.68	4	0.9207			
Cor Total	43.26	7				
*R* ^2^	Predicted *R*^2^		Adjusted *R*^2^		Adeq Precision	
0.9149	0.6595		0.8510		11.1891	

**Table 3:** Properties of various CA gel preparations in comparison with the plain gel.

Parameter	Plain Carbopol Gel	Free CA Gel	CA Novasomal Gel
Homogeneity	Homogeneous	Homogeneous	Homogeneous
Spreadability (mm)	38.67 ± 1.15	41.33 ± 0.58	36.33 ± 1.53
pH	5.25 ± 0.08	5.65 ± 0.16	5.59 ± 0.10
Drug content (%)	–	99.26 ± 1.06	98.97 ± 0.46

**Table 4:** Rheological properties of various gel preparations.

Rheological Properties	Plain Carbopol Gel	Free CA Gel	CA Novasomal Gel
Viscosity at 49 rpm (cP)	2770 ± 35	1583 ± 95	2833 ± 38
Viscosity at high shear rate (cP)	1035 ± 15	655 ± 48	1145 ± 8.6
Viscosity at low shear rate (cP)	11,200 ± 346	6600 ± 1039	12,800 ± 346
Yield stress (D/cm^2^)	4065.3 ± 164	1841.6 ± 191	4683 ± 104
Viscosity coefficient (cP) (×10^7^)	14.00 ± 6.03	8.09 ± 3.51	26.61 ± 9.15
Farrow’s constant (n)	3.07 ± 0.02	3.11 ± 0.02	2.89 ± 0.03
Flow index	0.29 ± 0.002	0.37 ± 0.01	0.33 ± 0.002
Apparent viscosity (×10^4^)	10.22 ± 0.43	3.28 ± 0.36	9.64 ± 0.27
Confidence of fit (%)	94.2 ± 0.05	95 ± 0.7%	94.3 ± 0.2
Thixotropy (D/s.cm^2^) (×10^5^)	2.91 ± 0.29	1.11 ± 0.38	1.69 ± 2.8

**Table 5:** Ex vivo permeation parameters of various CA formulations through rat abdominal skin.

Preparation	% CA Permeated After 24 h	*J*_ss_ (µg/cm^2^)	*P*_app_ (cm/h) ^a^	%EF
CA suspension	60.91 ± 31	34.19 ± 0.54	3.42 ± 0.05	–
Free CA gel	35.29 ± 0.17	22.62 ± 0.37	22.62 ± 0.37	66.18 ± 1.51
CA novasomes	87.25 ± 0.41	46.51 ± 0.39	4.65 ± 0.04	136.08 ± 2.80
CA novasomal gel	63.67 ± 0.39	37.87 ± 0.17	3.79 ± 0.02	110.78 ± 2.11

^a^ The values are multiplied by 10^2^.

**Table 6:** Inhibition zones and MIC of tested CA formulations against *S. aureus* and *E. coli*.

Formulation	Inhibition Zone (mm)		MIC (mg/mL)	
	*S. aureus*	*E. coli*	*S. aureus*	*E. coli*
Plain gel	0	0	Not determined	
CA suspension	20.0 ± 1.0	22.0 ± 2.0	0.312	0.312
Free CA gel	19.0 ± 1.0	15.3 ± 0.6	Not determined	
CA novasomes	22.3 ± 1.5	27.0 ± 1.0	0.312	0.156
CA novasomal gel	23.0 ± 1.0	20.3 ± 1.5	Not determined	

**Table 7:** Independent and dependent variables of CA novasomes formulations according to 2^3^ factorial design.

Parameters	Level Used	
Independent variables	Low (−1)	High (+1)
X1: Oleic acid (mg)	30	60
X2: Type of surfactant	SP60	TW80
X3: Oleic acid: surfactant ratio	1:1	1:2
Responses	Goal	
Y1: EE%	Maximize	
Y2: PS (nm)	Minimize	
Y3: ZP (mV)	Maximize	

## Data Availability

All data generated or analyzed during this study are included in this published article.
